# The Response of Corneal Endothelial Cells to Shear Stress in an In Vitro Flow Model

**DOI:** 10.1155/2021/9217866

**Published:** 2021-11-27

**Authors:** Sujuan Duan, Yingjie Li, Yanyan Zhang, Xuan Zhu, Yan Mei, Dongmei Xu, Guofu Huang

**Affiliations:** ^1^Department of Ophthalmology, First Hospital of Nanchang, Nanchang, Jiangxi 330008, China; ^2^Jiangxi Key Laboratory of Cancer Metastasis and Precision Treatment, First Hospital of Nanchang, Nanchang, Jiangxi 330008, China; ^3^Medical Department of Graduate School, Nanchang University, Nanchang, Jiangxi 330006, China; ^4^Department of Ophthalmology, Xingguo People's Hospital, Ganzhou, Jiangxi 342400, China

## Abstract

**Purpose:**

Corneal endothelial cells are usually exposed to shear stress caused by the aqueous humour, which is similar to the exposure of vascular endothelial cells to shear stress caused by blood flow. However, the effect of fluid shear stress on corneal endothelial cells is still poorly understood. The purpose of this study was to explore whether the shear stress that results from the aqueous humour influences corneal endothelial cells.

**Methods:**

An in vitro model was established to generate fluid flow on cells, and the effect of fluid flow on corneal endothelial cells after exposure to two levels of shear stress for different durations was investigated. The mRNA and protein expression of corneal endothelium-related markers in rabbit corneal endothelial cells was evaluated by real-time PCR and western blotting.

**Results:**

The expression of the corneal endothelium-related markers ZO-1, N-cadherin, and Na^+^-K^+^-ATPase in rabbit corneal endothelial cells (RCECs) was upregulated at both the mRNA and protein levels after exposure to shear stress.

**Conclusion:**

This study demonstrates that RCECs respond favourably to fluid shear stress, which may contribute to the maintenance of corneal endothelial cell function. Furthermore, this study also provides a theoretical foundation for further investigating the response of human corneal endothelial cells to the shear stress caused by the aqueous humour.

## 1. Introduction

Mechanical signals can regulate various cellular biological behaviours, such as cell proliferation, apoptosis, migration, differentiation, and shape reconstruction [1–6]. A variety of tissue cells, such as vascular endothelial cells [7–9], smooth muscle cells [10, 11], stem cells [5, 12, 13], and leukocytes [14, 15], have been studied after exposure to mechanical stimuli. The importance of mechanical signals is particularly relevant to the cardiovascular system. Both corneal endothelial cells and vascular endothelial cells exist in similar fluid environments, but little is known about the effect of fluid on corneal endothelial cells.

The limited studies about the effects of fluid force on ocular tissues have shown that the shear stress generated by fluid flow can regulate cell morphology, gene expression, and signalling cascades. For example, Akihiro [16] demonstrated that shear stress regulated gene expression in human retinal microvascular endothelial cells. Ulrike et al. found that shear stress could change cellular morphology and affect cell junction protein expression [17]. High shear stress may disrupt the phenotype and barrier function of the blood barrier in the retina [18]. Fluid shear stress inhibited wound healing and may be associated with modulation of the TGF-*β* signalling pathway in cultured corneal epithelial cells [19]. Application of axial strain in the physiological range to rabbit corneal fibroblasts downregulated *a*-SMA expression [20]. Corneal epithelial cells responded favourably to shear stress and exhibited morphological alterations, which affected their proliferation and migration behaviours, in response to two different magnitudes of shear stress [21]. We speculated that shear stress that results from the aqueous humour may also influence corneal endothelial cells. Yasuaki et al. also found that when human corneal endothelial cells were exposed to a fluid environment, the number of cells attached to the slide decreased as the magnitude and duration of the shear stress increased [22]. However, there are currently no reports on the gene expression of corneal endothelial cells after exposure to shear stress.

In studies of the effects of fluid shear stress on the ocular surface, the main focus has been on the effects of fluid flow caused by blinking on the corneal epithelium. Only a few studies reported the magnitude of fluid shear stress to which the corneal endothelium is exposed [22, 23]. In fact, the exact magnitude of the shear stress to which corneal endothelial cells are exposed under physiological conditions in vivo is still unknown. Yasuaki calculated the magnitude of the shear stress to which corneal endothelial cells are exposed due to the aqueous humour using three-dimensional anterior chamber models and the magnitude of shear stress used by Yasuaki ranged from 0 to 0.58 dyn/cm [2, 22]. Yuichi proposed the porcine endothelial cells were exposed to shear stresses (0.1–10 dyn/cm^2^) for 15 minutes for the experimental conditions [23]. As reference materials, we investigated the response of RCECs to two levels of shear stress (0.5 dyn/cm^2^ and 2 dyn/cm^2^). In preliminary experiments, we compared the effects of exposure to 0.5 dyn/cm^2^ and 1 dyn/cm [2], and the there is no significant difference of genes expression (data not shown). Another reason is that we cannot generate shear stresses below 0.5 dyn/cm^2^ because of the limitations of the peristaltic pump. In the present study, we chose two levels of shear stress (0.5 dyn/cm^2^ and 2 dyn/cm^2^) to investigate the effect of fluid flow on RCECs. A complete parallel plate flow chamber system was established to generate fluid shear stress. Then, the following conditions were chosen: (1) 0.5 dyn/cm^2^ shear stress for 30 min or 2 h, (2) 2 dyn/cm^2^ shear stress for 30 min or 2 h, and (3) no shear stress (control group). We analysed responses of RCECs to shear stress, including changes in gene and protein expression.

## 2. Materials and Methods

### 2.1. Animals

Healthy New Zealand white rabbits weighing 2.0–3.0 kilograms were used in the present study and were purchased from the Department of Experimental Animal Science of Nanchang University (Jiangxi, China). All the animals used in the study were handled according to the Association for Research in Vision and Ophthalmology (ARVO) statement.

### 2.2. Isolation and Culture of Cells

The RCECs were isolated using the previously described “peel-and-digest” method with some modifications [24]. Briefly, rabbit eyes were obtained with a sterile surgical apparatus after the rabbits had been sacrificed. Under a stereoscopic dissecting light microscope, the whole cornea was dissected from the limbal zone, and Descemet's membrane, containing the endothelium, was then peeled off. Next, Descemet's membrane was washed with PBS three times and incubated in a basal culture medium (DMEM; with 10% foetal bovine serum and 1% penicillin-streptomycin) overnight at 37 °C in 5% CO_2_. To isolate the primary corneal endothelial cells, the membrane was washed with PBS again and then digested with 0.5 mL of 0.25% ethylenediaminetetraacetic acid (EDTA) (Solarbio, Beijing, China) for 2 min at 37 °C in 5% CO_2_. Then, the EDTA was carefully removed, and the cells were suspended by flushing with the basal culture medium. Then, a suspension (2 × 10^6^ cells per ml) was cultured in Corning 35 mm tissue culture dishes for the following experiments.

### 2.3. Shear Stress Experiments

**Figure 1:**
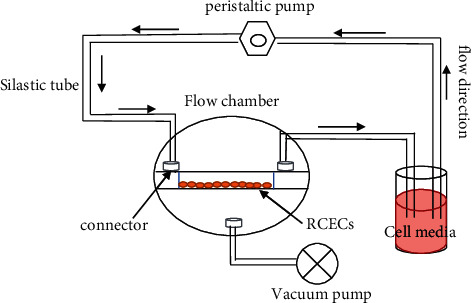
Diagram of the flow circuit system. The flow circuit included a parallel plate flow chamber, a peristaltic pump, a vacuum pump, and a medium reservoir, and these components were connected by silicone tubes and connectors.

A parallel plate flow chamber (Glycotech, Gaithersburg, MD, USA) was used to expose cell monolayers to laminar shear stress. One side of the flow chamber was the Corning 35 mm tissue culture dishes in which the RCECs were cultured, and the other side was the flow decks, which included three threaded holes to fit the inlet, outlet, and vacuum pump. These two flat surfaces were held apart by a round gasket. The flow chamber kit included a parallel plate flow chamber, a peristaltic pump (Baoding Longer Precision Pump Co., Ltd, China), a vacuum pump (Shanghai, China), and a medium reservoir. The vacuum pump tightly held the three components of the parallel plate flow chamber. These components were connected with several silicone tubes and connectors.  Figure 1 shows a schematic of the experimental setup used in the study. The shear stress acting on the cells was estimated using the following equation: ƮƮ = 6 *μ*Q/a^2^b, where Ʈ is the shear stress in dyn/cm [2], *µ* is the apparent viscosity of the perfused fluid, *Q* is the volumetric flow rate (mL/s), and a and *b* are the channel height (i.e., gasket thickness) and width (i.e., gasket width) [16, 25]. The shear stress was altered by changing the pump rotation velocity and volume flow. All the flow experiments were conducted in a standard incubator. Before applying shear stress to the RCECs, the Corning 35 mm tissue culture dishes were treated with 50 ug/ml fibronectin (BD, America) for 2 h in the incubator, and the same procedure was applied to the control group. Then, the fibronectin was removed, and 2 × 10^6^ cells per ml were seeded in the Corning 35 mm tissue culture dishes and grown until the cells reached confluence. Then, the cells were used for subsequent fluid experiments. The cells in the experimental group were exposed to shear stress, and cells from the same rabbit seeded in fibronectin-coated Corning culture dishes but not exposed to shear stress were used as controls.

### 2.4. Real-Time PCR

Total RNA was extracted from RCECs with TRIzol reagent (Tiangen Biotech, Beijing, China) according to the manufacturer's instructions. The genomic DNA in the samples was then removed with DNase, and then, the total RNA concentration of each sample was measured by using Nanodrop 2000 (Thermo, ND-2000). cDNA was synthesized in a 20 *μ*l reaction system containing 1 *μ*g of RNA with a cDNA Synthesis Kit (Takara, Dalian, China) according to the protocol. Then, real-time PCR was conducted in a 20 *μ*l reaction mix that included 10 *μ*l SYBR Green (Takara, Dalian, China), 0.5 *μ*l paired primers, 2 *μ*l cDNA, and 7.5 *μ*l ddH_2_O, and the cycling conditions were as follows: 95 °C for 20 s followed by 45 cycles of 94 °C for 10 s and 58 °C for 35 s. Relative gene expression was analysed using the comparative Ct (ΔΔCt) method. The primers used in real-time PCR are listed in Table 1.

### 2.5. Western Blotting

RCECs stimulated with or without shear stress were collected using a scarper and subsequently lysed with RIPA buffer containing protease inhibitors according to the manufacturer's protocol. The lysates were centrifuged, and the supernatants, which contained the total proteins, were then collected. Protein quantification was performed with a BCA Protein Assay Kit (Beyotime, Shanghai, China) according to the manufacturer's protocol. To denature the proteins, 20 *μ*g of proteins and 5X sodium dodecyl sulfate (SDS) were mixed together and boiled for 10 minutes. Then, the proteins were loaded onto a 10% sodium dodecyl sulfate‐polyacrylamide gel electrophoresis (SDS‐PAGE) gel, separated by electrophoresis, and then transferred to a polyvinylidene fluoride (PVDF) membrane (Millipore Corp, Bedford, MA). Then, the membrane was blocked with 5% skim milk at room temperature for 2 h, followed by incubation with the primary antibody at 4 °C overnight. Next, the membrane was incubated with the corresponding secondary antibody. The protein bands were detected using an enhanced chemiluminescent reagent (KeyGEN BioTECH, Nanjing, China), and the band densities were analysed by ImageJ.

### 2.6. Statistics

All the experiments in the study were repeated at least three times. All the values are represented as the mean ± standard error. One-way ANOVA was used to compare the mean values, and the statistical significance of differences was determined using Bonferroni's post hoc test when a significant F ratio was observed. *P* < 0.05 was considered statistically significant.

## 3. Results

### 3.1. The mRNA Expression of Corneal Endothelial Cell-Related Markers in Response to Shear Stress

**Figure 2:**
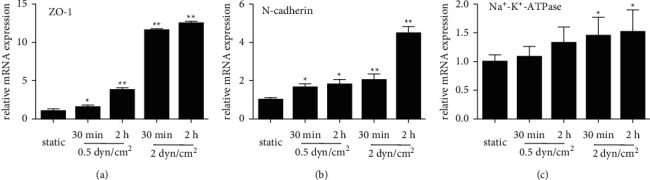
Effect of shear stress on the mRNA expression of corneal endothelial cell-related markers. (a) ZO-1 mRNA expression was significantly increased after treatment with shear stress (0.5 dyn/cm^2^ and 2 dyn/cm^2^) for 30 min and 2 h. (b) N-cadherin mRNA expression gradually increased as the shear stress intensity increased compared with the static control. (c) Na^+^-K^+^-ATPase mRNA expression was slightly increased after exposure to shear stress. The data are presented as the mean ± SD; ^*∗*^*P* < 0.05, ^*∗∗*^*P* < 0.01.

The expression levels of corneal endothelial cell-related genes were evaluated under shear stress and static conditions.  Figure 2 shows the altered mRNA expression of the corneal endothelial cell-related markers ZO-1, N-cadherin, and Na+-K+-ATPase in RCECs cultured under static and shear stress conditions. After the continuous exposure of RCECs to 0.5 dyn/cm^2^ for 0.5 h and 2 h, ZO-1 mRNA expression gradually increased with prolonged exposure time compared with that in the cells exposed to static control conditions (1.55 ± 0.276 and 3.76 ± 0.29 vs. 1 ± 0.32). When the cells were exposed to shear stress up to 2 dyn/cm [2], the mRNA expression of ZO-1 was significantly upregulated (11.6 ± 0.23 and 12.24 ± 0.31 vs. 1 ± 0.32) (Figure 2(a)). The mRNA levels of N-cadherin showed a trend similar to that of ZO-1 mRNA expression after exposure to shear stress (Figure 2(b)). The mRNA expression of Na^+^-K^+^-ATPase was slightly increased under lower stress conditions. When shear stress reached 2 dyn/cm [2], the expression levels of Na^+^-K^+^-ATPase were significantly upregulated (1.45 ± 0.33 and 1.52 ± 0.38 vs. 1 ± 0.12) (Figure 2(c)).

### 3.2. Effects of Shear Stress on the Expression of Proteins Related to Corneal Endothelial Cell Function

**Figure 3:**
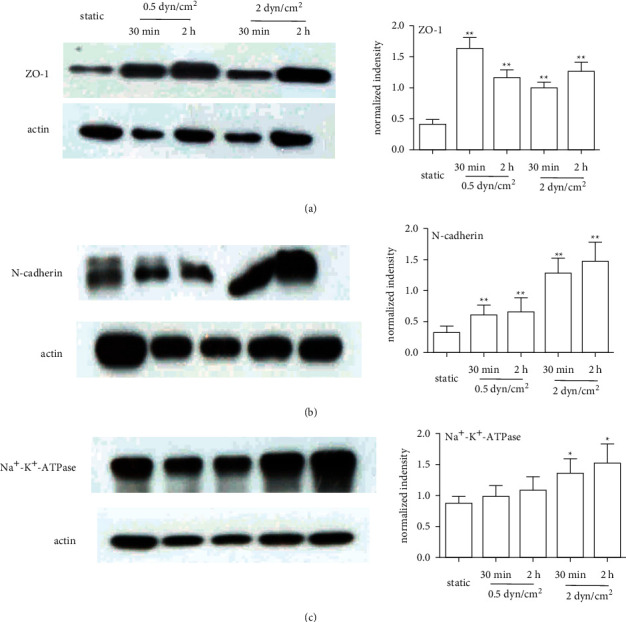
Effect of shear stress on the protein expression of corneal endothelial cell-related markers. (a) ZO-1 protein expression was significantly increased in the shear stress groups compared with the static group. (b) N-cadherin protein expression gradually increased with increasing shear rates and exposure time. (c) Na^+^-K^+^-ATPase protein expression was slightly increased after exposure to shear stress. All the experimental data were analysed in triplicate; ^*∗*^*P* < 0.05, ^*∗∗*^*P* < 0.01.

The protein levels of corneal endothelial cell-related markers were assessed in at least three independent experiments. ZO-1 protein expression was significantly increased under shear stress conditions compared to static conditions (Figure 3(a)). This effect did not seem to occur in a time- or force-dependent manner. The protein expression of both N-cadherin and Na^+^-K^+^-ATPase gradually increased with increasing shear force and exposure time, which appeared to be consistent with the mRNA expression results described above (Figures 3(b) and 3(c)).

## 4. Discussion

Corneal endothelial cells (CECs) form a monolayer of hexagonal cells between Descemet's membrane (DM), and the aqueous humour serves as a barrier and a pump, thus playing a pivotal role in the maintenance of corneal transparency [26]. The aqueous humour, a fluid circulating in the eye, plays a crucial role in maintaining the homeostasis of corneal endothelial cells. Although the influence of flow on vascular endothelial cells has been well documented, fluid flow within corneal endothelial cells has been ignored. We, therefore, hypothesized that shear stress from the aqueous humour may also exert meaningful effects on corneal endothelial cells. The present study demonstrated, for the first time, that fluid shear stress affected the gene expression of corneal endothelial cell-related markers in cultured RCECs.

A major function of the corneal endothelium is to maintain corneal transparency by regulating corneal hydration, which is mediated by its barrier and pump function [27, 28]. ZO-1 is a tight junction protein, and N-cadherin is an anchoring junction protein. Both proteins play key roles in barrier function and maintain the water content in the cornea at a suitable level [29–31]. Na^+^/K^+^-ATPase, a membrane transport protein located in the corneal endothelium, promotes the pump function of corneal endothelial cells by removing excess stromal fluid to maintain corneal transparency [32, 33]. Shear stress can be beneficial to maintain the function of different types of cells, depending on the magnitude and duration of the shear stress [34–36]. In the current study, corneal endothelial cells exhibited altered transcription levels and protein expression levels of marker genes in response to the application of shear stress (2 and 3). The mRNA levels of ZO-1 were significantly upregulated in the 2 dyn/cm^2^ group, whereas the protein expression levels were slightly altered in our study. The inconsistent results may be due to the influence of multiple factors, and mRNA changes do not necessarily always correlate with protein changes. Overall, shear stress induced the expression of corneal endothelial cell function-related markers, which is consistent with previous studies performed on vascular endothelial cells [37–39]. These results reveal that RCECs are sensitive to shear stress, which may be involved in the maintenance of corneal endothelial cell function.

This study had some limitations. First, we just observed the alterations of the cell morphology under the microscope with or without shear stress exposure. As shown in Supplementary Figure 1, there is no significant change in cell morphology after shear stress treatment. This is inconsistent with the research proposed by Akihiro and Nicole that the cells are aligned with the direction of the fluid after shear stress exposure [16, 40]. We speculated that the reasons were related to the different cell types and short time of shear stress exposure. But, when we prolonged the RCEC exposure time, more cells started to detach from the culture dishes, even though we changed the perfusion medium, which is consistent with the literature reports [22, 23]. We also conducted ZO-1 staining analysis. However, it has encountered obstacles for us since RCECs exposed to shear stress are easier to get detached from the slides during the process of fixing and washing. We hope to obtain better results by optimizing the experimental conditions in the next experiments. Second, when applying the in vitro data to in vivo situations, some differences should be considered; for example, the attachment of the cells to culture slides or to Descemet membranes may not be the same. Our experimental device is a closed circuit that consists of only corneal endothelial cells and unidirectional laminar flow. This might explain why shear stress increased the expression of corneal endothelial cell-related genes. In fact, corneal endothelial cells are influenced by the shear stress of the aqueous humour, various growth factors in the aqueous humour, the biophysical microenvironment of Descemet's membrane, hydrostatic intraocular pressure, and the temperature of the cornea [41–44]. We cannot simulate the combined effects of these factors on corneal endothelial cells in the present study. Thus, we need to interpret the results of the current study with caution. Third, the mechanism by which increased expression of corneal endothelial cell-related markers was induced after shear stress has not been elucidated, but the mechanism may be related to the fluid activating-related intracellular signalling pathways and requires further investigation. Last, we did not replicate our results in human corneal endothelial cells. Although human corneal endothelial cells are more difficult to expand in vitro when compared with rabbit corneal endothelial cells, a large number of studies have reported on the method of promoting the proliferation of human corneal endothelial cells in vitro in recent years [45–48]. Our group's ongoing experiments need to be replicated in human corneal endothelial cells to explore the involved mechanisms.

In conclusion, the results of our study demonstrated for the first time that the shear stress of the aqueous humour upregulated the mRNA and protein expression levels of ZO-1, N-cadherin, and Na^+^/K^+^-ATPase in RCECs. This effect suggested that physiological levels of fluid shear stress may contribute to the function of corneal endothelial cells, and this study lays a foundation for further related research in the future.

## Figures and Tables

**Table 1:** The primers used for real-time PCR.

GENE	Forward Primer	Reverse Primer
ZO-1	AGTTTGGCAGCAAGAGATGG	GCTGTCAGAAAGGTCAGGGA
Na^+^/K^+^-ATPase	CGGCTACAAAGACGGCAAAC	GAACAGGCAGCACATTTGGG
N-cadherin	ATGGCTTGGAATGAGACTGC	CCACCAGAGTGAAAGGAACG
GAPDH	GCACCGTCAAGGCTGAGAAC	TGGTGAAGACGCCAGTGGA

## Data Availability

The datasets used during the current study are available from the corresponding author upon reasonable request.

## Supplementary Materials

Supplementary Materials S1 Figure 1: morphological changes in RCECs after exposure to shear stress were observed under the microscope. (DOCX).
