# Supplementary material for: The Response of Corneal Endothelial Cells to Shear Stress in an In Vitro Flow Model
Source: J Ophthalmol. 2021 Nov 27;2021:9217866. doi: 10.1155/2021/9217866 (PMC8643247; doi:10.1155/2021/9217866)
Supplement: Supplementary Materials — S1 Figure 1: morphological changes in RCECs after exposure to shear stress were observed under the microscope. (DOCX). [file 9217866.f1.docx]

**Supplementary materials**

**Effect of shear stress on morphological alterations in RCECs**

RCECs cultured on the Corning culture dishes were exposed to different magnitude of shear stress (0 and 2dyn/cm2) for 2hours and then the cells were observed under the microscope. As shown in figure 1, the cells of a confluent monolayer appeared to be polygonal and randomly disposed under the static condition (0 dyn/cm^2^). Under flow conditions (2 dyn/cm^2^), the cells did not appear to align with flow and there is no significant change in cell morphology after shear stress treatment.
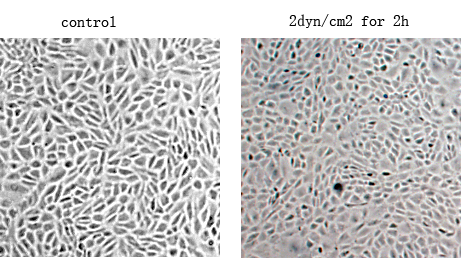


**supplementary Figure 1. Morphological changes in RCECs after exposure to shear stress were observed under the microscope.** (A)RCECs without shear stress exposure as static control. (B) RCECs exposed to 2 dyn/cm2 shear stress for 2h.
